# What should General Adult Psychiatrists know about Reproductive Counselling of Women with Severe Mental Illness?

**DOI:** 10.1192/j.eurpsy.2022.170

**Published:** 2022-09-01

**Authors:** T. Kurimay

**Affiliations:** North Centre Buda New Saint John Hospital and Oupatient Clinic, Buda Family Centre Mh Centre Department Of Psychiatry, Teaching Dep. Of Semmelweis University, Budapest, Hungary

**Keywords:** risk management, planning pregnancy, consequences of untreated mental illness, interdisciplinary and family members collaboration

## Abstract

A notable part of psychiatry is prevention. Our job, as psychiatrists, is not only to treat mental disorders, but also to prevent them. Treating mothers in postnatal period reducing the negative consequence of depression for child development, for example. General adult psychiatrists face the challenge of having patients, who receive psychiatric health care, while also planning to have a child. Many professionals may find themselves in a situation, when they feel clueless on what to advise regarding pharmacotherapy and realistic expectations on having a child. The presentation will focus on some crucial topics. What should counselling include when planning pregnancy, highlighting differences among first and second or further children. Potentials risks and harms on the fetus / new-born baby will be introduced with emphasis on pharmacological/chemical agents, infectious effects and social, relational and family stressors. What the guidelines are for Covid-19 vaccination and pregnancy. Relative and absolute contraindications of planned pregnancy will be discussed. The crucial question of artificial/therapeutic abortion; are there any psychiatric conditions, when a psychiatrist can/should suggest it? The advantage and knowledge of perinatal mental health guideline papers, bio-ethical aspects will be discussed, along with the consequences of untreated mental illness. Advantage of breastfeeding and an up-to-date view on what should be psychiatrists’ aim will be introduced. Why is it inevitable to cooperate with GPs, obstetric- and gynecology colleagues and further medical professionals? The essential aspect and advantages of involving fathers and extended family members in this approach also will be explained.

**Disclosure:**

No significant relationships.

